# Immunomodulatory activity of SGI-110: a basis for novel chemo-immunotherapeutic combinations in cancer treatment

**DOI:** 10.1186/2051-1426-1-S1-P71

**Published:** 2013-11-07

**Authors:** Alessia Covre, Giulia Parisi, Hugues JMG Nicolay, Carolina Fazio, Ester Fonsatti, Elisabetta Fratta, Luca Sigalotti, Pietro Taverna, Gavin Choy, Mohammad Azab, Sandra Coral, Michele Maio

**Affiliations:** 1 Division of Medical Oncology and Immunotherapy, Department of Oncology, University Hospital of Siena, Siena, Italy; 2 Cancer Bioimmunotherapy Unit, Centro di Riferimento Oncologico, IRCCS, Aviano, PN, Italy; 3 Astex Pharmaceuticals Inc., Dublin, CA, USA

## 

Aberrant DNA hypermethylation favors tumor escape from host’s immune recognition by decreasing the expression of tumor-associated antigens (TAA) (e.g., cancer testis antigens (CTA)), HLA, co-stimulatory molecules that are all required for efficient immune recognition of cancer cells. Thus, increased levels of DNA methylation in cancer cells might contribute to reduced clinical efficacy of immunotherapeutic approaches. SGI-110 is a dinucleotide of decitabine (DAC) and deoxyguanosine formulated as a low volume SQ injection which in the clinic, provides more extended DAC exposure compared to DAC IV. The observed superior PK profile offers the potential of improved biological and clinical activity with a better safety profile compared to currently available hypomethylating agents. We recently demonstrated that SGI-110 induced/up-regulated CTA expression in cancer cells of different histotype at both mRNA and protein levels. Quantitative methylation-specific PCR analyses identified hypomethylation of MAGE-A1 and NY-ESO-1 promoters in SGI-110-treated cancer cells, demonstrating a direct role of pharmacologic DNA demethylation in CTA expression. SGI-110 also up-regulated the expression of HLA class I and ICAM-1, resulting in an improved recognition of cancer cells by TAA-specific CTL. These immunomodulatory properties of SGI-110 are further supported by in vivo findings with human melanoma xenografts. Furthermore, preliminary data, generated in a syngeneic model of murine cancer, demonstrate the synergistic anti-tumor effectiveness of SGI-110 when administered in combination with immunostimulatory antibodies (i.e., anti-CTLA-4 and -PD-1). These findings were extended to the clinical setting, where the hypomethylating activity of SGI-110 on CTA-specific promoters and the resulting induction/up-regulation of NY-ESO-1, MAGE-A1, MAGE-A3 expression were characterized in PBMC from MDS or AML patients. Together, these preclinical and clinical data suggest that SGI-110 in addition to having direct anti-tumor activity as a single agent may sensitize tumor cells to agents acting through the immune system and hence it may increase their clinical activity. These evidences, together with the favorable pharmacologic and pharmacokinetic features of SGI-110, provide a strong scientific rationale to develop new anti-cancer therapies based on the combined efficacy of SGI-110 and immunotherapeutic strategies.

